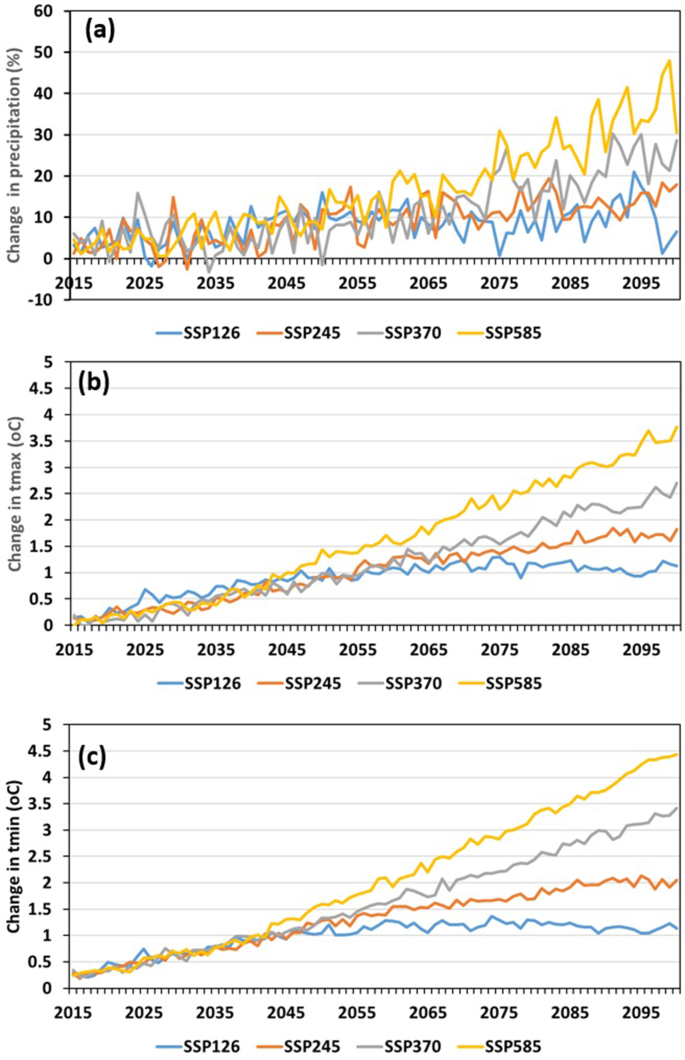# Predicted changes in future precipitation and air temperature across Bangladesh using CMIP6 GCMs

**DOI:** 10.1016/j.heliyon.2023.e16274

**Published:** 2023-05-13

**Authors:** Mohammad Kamruzzaman, Shahriar Wahid, Shamsuddin Shahid, Edris Alam, Mohammed Mainuddin, H. M. Touhidul Islam, Jeapil Cho, Md Mizanur Rahman, Jatish Chandra Biswas, Kelly R. Thorp

**Affiliations:** a Farm Machinery and Postharvest Technology Division, Bangladesh Rice Research Institute, Gazipur, 1701, Bangladesh; b CSIRO Environment, Black Mountain Laboratories, Canberra, ACT, Australia; c Universiti Teknologi Malaysia (UTM), 81310, Johor, Malaysia; d Rabdan Academy, Abu Dhabi, United Arab Emirates; e Department of Geography and Environmental Studies, University of Chittagong, Chittagong, Bangladesh; f CSIRO Environment, Canberra, ACT, Australia; g Department of Disaster Management, Begum Rokeya University, Rangpur, 5400, Bangladesh; h Convergence Center for Watershed Management, Integrated Watershed Management Institute (IWMI), Republic of Korea; i Krishi Gobeshona Foundation (KGF), BARC, Dhaka, Bangladesh; j USDA-ARS, Arid Land Agricultural Research Center, Maricopa, AZ, 85138, United States

**Keywords:** Global climate models, Multi-model ensemble, Shared socioeconomic pathways, Downscaling, Projections

## Abstract

Understanding spatiotemporal variability in precipitation and temperature and their future projections is critical for assessing environmental hazards and planning long-term mitigation and adaptation. In this study, 18 Global Climate Models (GCMs) from the most recent Coupled Model Intercomparison Project phase 6 (CMIP6) were employed to project the mean annual, seasonal, and monthly precipitation, maximum air temperature (Tmax), and minimum air temperature (Tmin) in Bangladesh. The GCM projections were bias-corrected using the Simple Quantile Mapping (SQM) technique. Using the Multi-Model Ensemble (MME) mean of the bias-corrected dataset, the expected changes for the four Shared Socioeconomic Pathways (SSP1-2.6, SSP2-4.5, SSP3-7.0, and SSP5-8.5) were evaluated for the near (2015–2044), mid (2045–2074), and far (2075–2100) futures in comparison to the historical period (1985–2014). In the far future, the anticipated average annual precipitation increased by 9.48%, 13.63%, 21.07%, and 30.90%, while the average Tmax (Tmin) rose by 1.09 (1.17), 1.60 (1.91), 2.12 (2.80), and 2.99 (3.69) °C for SSP1-2.6, SSP2-4.5, SSP3-7.0, and SSP5-8.5, respectively. According to predictions for the SSP5-8.5 scenario in the distant future, there is expected to be a substantial rise in precipitation (41.98%) during the post-monsoon season. In contrast, winter precipitation was predicted to decrease most (11.12%) in the mid-future for SSP3-7.0, while to increase most (15.62%) in the far-future for SSP1-2.6. Tmax (Tmin) was predicted to rise most in the winter and least in the monsoon for all periods and scenarios. Tmin increased more rapidly than Tmax in all seasons for all SSPs. The projected changes could lead to more frequent and severe flooding, landslides, and negative impacts on human health, agriculture, and ecosystems. The study highlights the need for localized and context-specific adaptation strategies as different regions of Bangladesh will be affected differently by these changes.

## 1 Introduction

Climate change (CC) is currently the most pressing environmental concern of the twenty-first century. Its impact on food security is already evident, with changes in precipitation patterns, rising air temperatures, and more recurring extremes [1,2]. This issue is compounded by inadequate management of environmental resources and a limited ability to adapt in developing countries [3,4]. Bangladesh, as a developing country, is highly exposed to climatic shifts. They experienced a substantial rise in air temperature, monsoon and post-monsoon precipitation, while winter precipitation decreased during the late twentieth century due to CC [[5], [6], [7]]. Extreme weather events directly attributable to climatic changes have become increasingly common in recent years and have been responsible for substantial financial losses and human casualties in Bangladesh [8,9]. As global warming continues, extreme weather trends are projected to continue throughout the present century [[10], [11], [12]]. Thus, knowing how precipitation and air temperature will alter is vital to develop effective strategies for reducing climatic risk.

The Global Climate Models (GCMs) are the fundamental tool for understanding the potential impacts of CC [13,14]. Previous studies on the future climate of Bangladesh have mostly used GCMs from CMIP3 [15,16] and CMIP5 [7,10,11,[17], [18], [19]]. The Coupled Model Intercomparison Project phase 6 (CMIP6) GCMs based on CMIP6 are improved forms of preceding CMIPs in a number of ways, including better geographical resolution and improved modelling of cloud microphysical processes [20,21]. Therefore, the CMIP6 GCM ensemble is more reliable for climate projections than the previous CMIP ensembles.

GCMs contain systematic biases related to the observations [22]. Bias removal of GCM outputs is needed to study local- and regional-scale CC impacts on different sectors [[23], [24], [25]]. Dynamical and statistical techniques are used for downscaling and bias correction of GCM predictions. Statistical downscaling techniques develop statistical relations between reference datasets (Observed) and GCM outputs. In contrast, dynamical techniques involve a regional climate model (RCM) embedded within a GCM to produce high-resolution climate data [26]. Each technique has some benefits and shortcomings. The key disadvantages of dynamic downscaling strategies are high computing costs, data storage and errors [27]. Consequently, statistical methods are extensively employed in CC impact and adaptation research.

A wide range of techniques are used for correcting GCM biases, including quantile mapping [25], delta change [28], and mean correction [29]. Amongst them, quantile mapping methods are widely recognized as the most reliable [[30], [31], [32]]. This research used a quantile-based technique to correct the biases. Additionally, it is challenging to manage substantial uncertainty in GCM predictions. Climate projections are prone to three main types of uncertainty: future emissions (uncertainty associated with scenarios), internal climatic variability, and inter-model differences. Internal variability is essentially stable throughout time series, but other uncertainties increase with time but at varying rates. Different methodologies have yielded similar results, indicating no ideal way to separate the uncertainty [33]. Although various approaches have been suggested to deal with the uncertainty of climate projections, model ensembling is being most widely suggested to solve this problem and improve projection accuracy and reliability [29,34].

CC is a growing concern worldwide, leading to increased research on climate parameters and elements. Researchers have predicted changes in temperature and precipitation using GCMs in various regions. Numerous studies have investigated future CC using CMIP6 GCMs [[35], [36], [37], [38]]. In India, Sarkar et al. [39] found that precipitation is expected to decrease by 9–27% in future periods. Summer rainfall in Korea is forecast to drop while becoming more intense, according to research [40]. Meanwhile, Leong Tan et al. [41] evaluated the effects of climate change on Malaysia's water resources and found that the country can anticipate more precipitation during the wetter months and fewer during the drier months.

Summer rainfall in the southeastern United States has been shown to rise significantly under the influence of rising temperatures and water vapour flow, according to research Ferreira et al. [42]. According to the HadGEM2 model predictions for the Urmia Lake basin in northwestern Iran by Heydari et al. [43], precipitation is expected to decrease while temperature increases in the near future. Jiang et al. [44] projected a substantial increase in average annual rainfall for Central Asia by the end of the 21st century based on 15 models from the CMIP6 under four SSP scenarios. Finally, Yue et al. [38] examined changes in temperature and precipitation in the Yangtze River Basin in China and concluded that they will probably increase in the long term under different SSP scenarios, with some degree of uncertainty surrounding the exact values predicted.

Qin et al. [45] found better simulation for projecting temperature changes using five CMIP6 models than precipitation changes for northwest China. Regardless of the scenario, significant increases in both temperature and precipitation are expected in the 21st century. You et al. [46] utilized 20 GCMs from CMIP6 and three SSPs scenarios to forecast temperature changes in China. They determined that temperature is expected to increase in all future periods.

Progress has also been made in projecting the climate of Bangladesh [21,[47], [48], [49]]. Kamruzzaman et al. [48,49] assessed the relative performance of CMIP6 models. Kamal et al. [47] used six CMIP6 GCMs to estimate changes in precipitation and drought in Bangladesh for the two shared socioeconomic pathways (SSP1-1.9 and SSP1-2.6). Recently, Das et al. [21] assessed extreme rainfall characteristics using four CMIP6 GCMs for two emission scenarios. Since the simulation results depend on the models employed, it is more insightful to investigate changes in air temperature and precipitation in Bangladesh from a large set of models and diverse scenarios. The spatial and temporal trends and variability in future precipitation and temperature changes on seasonal and annual timescales are poorly understood. Moreover, none of the previous studies used a wide range of CMIP6 models and scenarios for projecting Bangladesh's climate. The present study is expected to address this gap.

The primary objective of this work was to shed light on the future of rainfall, maximum temperature (Tmax), and minimum temperature (Tmin) in Bangladesh using the most recent CMIP6 dataset. The aims of this study were to (1) assess how well CMIP6 models reproduced observed patterns of precipitation and temperature; (2) investigate the spatiotemporal variability and changes in projected precipitation and temperature; and (3) investigate the seasonal and annual precipitation, and temperature trends during the near (2015–2044), mid (2045–2074), and far (2075–2100) futures in Bangladesh. This study examined the annual and seasonal change in precipitation, Tmax, and Tmin only as they are the most critical climate variables for studying climate change impacts assessment and adaptation planning.

## 2 Study region and facts

### 2.1 Bangladesh

**Fig. 1:**
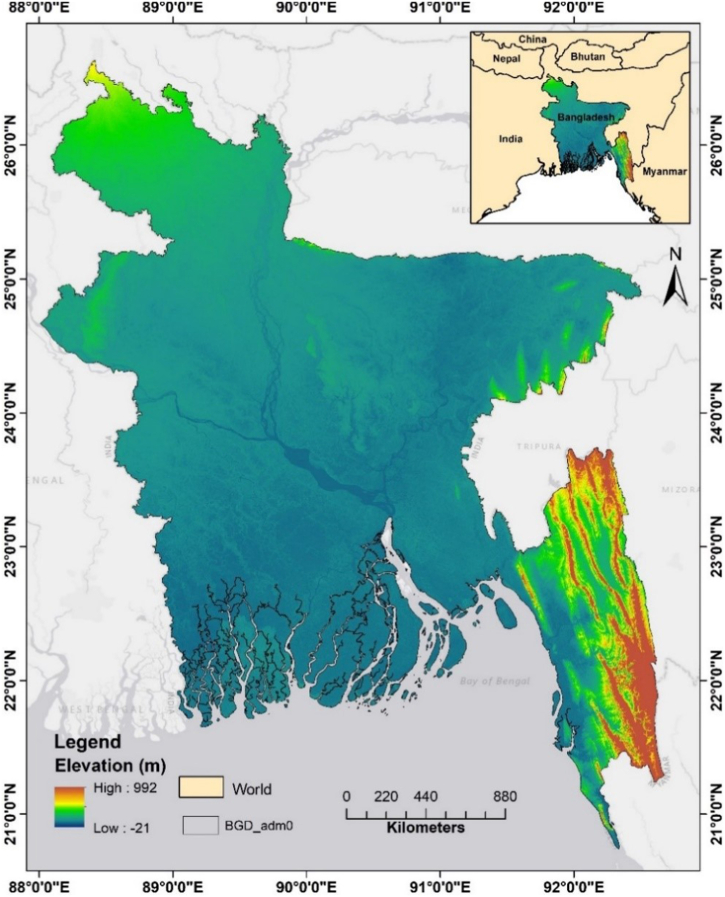
Topographic map of the study area. The elevation expressed in meters above means sea level. Source: Digital Elevation Model (DEM) from https://earthexplorer.usgs.gov/.

Bangladesh, a country in south Asia, is located between 20°34′N and 26°38′N and 88°01′E and 92°41′E (Fig. 1). With the exception of the hilly regions in the southeast and east, most of the 1,48,460 km^2^ land of the country consists of flood plains. There are low-lying areas with “delta-shaped” landforms due to elevation ranges 1–60 m above mean sea level [50]. Bangladesh experiences four distinct seasons, which are as follows: winter (December–February), pre-monsoon (March–May), monsoon (June–September), and post-monsoon (October–November), as stated by Jerin et al. [51]. The winter is very dry, whereas the monsoon season receives the vast bulk of its annual precipitation. Northwest-to-northeast rainfall ranges from 1500 to 4000 mm [52]. The highest summertime temperatures typically range from around 38 to about 41 °C. April is the hottest month while January is the coldest (with mean daytime highs of 16–20 °C and lows of approximately 10 °C throughout the country). Evapotranspiration is 3.72 mm d^−1^ and the mean daily relative humidity is 80% [9]. Bangladesh is often hit by a variety of natural catastrophes, including but not limited to floods, cyclones, and droughts. This makes it highly vulnerable to disasters and CC.

### 2.2 Data

#### 2.2.1 Reference data

There are numerous temporal and spatial inconsistencies in the climate records of Bangladesh. A scant number of weather stations are dispersed unevenly across the country. Additionally, weather stations are infrequent in remote areas and hilly mountainous regions. Grid-based climatic variables data for Bangladesh can be obtained from satellites and global model reanalysis. Based on rainfall and temperature detection metrics, ERA5 demonstrated superior performance in Bangladesh [53]. The ERA5 dataset was used by Zhai et al. [54] for predicting droughts in south Asian countries, including Bangladesh. Recently, Kamruzzaman et al. [49] used the ERA5 as reference data to evaluate the skills of CMIP6 GCMs in modeling rainfall data over Bangladesh. Therefore, we employed the ERA5 reanalysis as a reference dataset in this investigation. The climate data for ERA5 was retrieved from the ECMWF website (https://www.ecmwf.int/en/forecasts/datasets/reanalysis-datasets/era5). The ERA5 was developed using a technique to interpolate in-situ precipitation datasets, which included infrared cloud datasets as a covariate. The present study employed an ERA5 dataset covering the years 1985–2014, with a spatial resolution of 0.25°.

#### 2.2.2 Global Climate Models (GCMs)

The Earth System Grid (ESG) Data Portal (https://esgf-node.llnl.gov/search/cmip6) was used to gather the simulation results of 18 CMIP6 GCMs [14]. The GCMs were selected considering the availability of precipitation, Tmax, and Tmin simulations for SSP1-1.26, SSP2-4.5, SSP3-7.0, and SSP5-8.5. While there is no universally agreed upon “best performing” GCM, the selected models are widely used in climate modeling research and have been shown to produce reliable results in previous studies. The CMIP6 future scenario experiments are divided into main priority categories, including 1) the tier-1 experiments with SSPs (SSP1-1-2.6, SSP2-4.5, SSP3-7.0, and SSP5-8.5), and 2) the tier-2 experiments with SSPs (SSP1-1.9, SSP4-3.4, SSP4-6.0, and SSP5-3.4) [55]. The key features of each GCM, such as their developing institution and spatial resolution, are displayed in Table S1. This study only used the results from the first realization (r1i1p1f1) of each GCM to keep the evaluations consistent and to minimize the model bias. Considering the variable spatial resolutions among the models, a bilinear interpolation technique was used to re-grid the GCM historical simulations to the ERA5 grid resolution (0.25° × 0.25°) for the assessment. Recently, Kamruzzaman et al. [49] used an analogous method to evaluate CMIP6 GCMs in Bangladesh.

## 3 Methods

### 3.1 Bias-correction

Daily GCM simulations for the years 1985–2100 were downscaled to ERA5 resolution using the SQM method and then bias-corrected based on ERA5 data. The SQM approach uses empirical quantile mapping to refine GCM simulations independently. The following three-steps were: (1) extraction of the GCM data corresponding to each target ERA5 grid location, (2) assessment of GCM's biases, and (3) bias-correction of projections. Using Eq. (1), the retroactive period differences between observed and simulated cumulative distribution functions (CDFs) were calculated and applied to future simulations for a particular percentile:

(1) $$x_{p}^{\prime }\left(t\right){=x}_{p}\left(t\right)+F_{obs}^{-1}\left(F_{p.sim}\left(x_{p}\left(t\right)\right)\right)-F_{r.sim}^{-1}\left(F_{p.sim}\left(x_{p}\left(t\right)\right)\right)$$

where GCM bias-corrected and raw predictions for day t are denoted by *x′p*(t) and *xp*(t), respectively. *F(θ)* and *F*^*−1*^*(θ)* are a CDF of GCM simulations, θ, and its inverse, respectively. Subscripts *p.sim*, *r.sim*, and *obs* denote the projection, retrospective simulation, and ERA5 daily data, respectively [7]. ERA5 and raw GCM data are used as inputs in an empirical equation that is not parametric because such approaches have been shown to be more effective at minimizing systematic bias than parametric methods [56].

### 3.2 Performance evaluation

A graphical tool known as the Taylor Diagram is commonly used to present the performance of a model using a range of metrics [57]. It offers a concise overview of the statistical associations among the observations and models by depicting the root mean square (RMS) difference, correlation coefficient (R), and the ratio of standard deviations (SDs) of two patterns on a single graph [57].

Root Mean Square Deviation (RMSD): RMSD is a measure of the difference between predicted and observed values. It is often used to evaluate the accuracy of mathematical models or simulations. RMSD can be calculated by Eq. (2).

(2) $$RMSD=\sqrt{\frac{1}{n}}\sum {\left(y\_pred-y\_obs\right)}^{2}$$

where: n is the number of observations, y_pred is the predicted value, y_obs is the observed value or true value. Σ is the summation operator that sums over all observations.

Standard Deviation: Standard deviation is a measure of the amount of variation or dispersion in a set of data. It is often used to describe the spread of a data set around its mean. Standard deviation (σ) can be calculated by Eq. (3).

The formula for standard deviation is:

σ = √(Σ(x_i - μ)^2^ / (n - 1))

(3) $$\sigma =\sqrt{(\sum \frac{{\left(x_{i}-\mu \right)}^{2}}{\left(n-1\right)}})$$

where: σ is the sample standard deviation. x_i is the i-th observation in the sample. μ is the sample mean. n is the sample size. Σ is the summation operator that sums over all observations.

Correlation Coefficient: Correlation coefficient is a statistical measure that indicates the degree of linear relationship between two variables. Correlation Coefficient (r) can be calculated by Eq. (4).

(4) $$r=\frac{\sum \left(\left(x\_i-\mu \_x\right)\left(y\_i-\mu \_y\right)\right)}{(\sqrt{\sum {\left(x\_i-\mu \_x\right)}^{2}}\;\sqrt{\left(\sum {\left(y\_i-\mu \_y\right)}^{2}\right))}}$$

where: r is the Pearson correlation coefficient, x_i is the i-th observation in the x dataset, y_i is the i-th observation in the y dataset. μ_x is the mean of the x dataset, μ_y is the mean of the y dataset, Σ is the summation operator that sums over all observations.

The numerator calculates the sum of the product of the deviations of x and y from their respective means, while the denominator is the product of the standard deviations of x and y. This formula is used to measure the linear relationship between two variables, x and y, ranging from −1 (perfect negative correlation) to 1 (perfect positive correlation).

The simulation of the model is optimal when RMS is nearer to 0, and R and the ratio of SDs are equivalent to 1. The ability of both the uncorrected and bias-corrected models was assessed using the Taylor Diagram. Previous studies have used the Taylor diagram to evaluate the GCMs [38,48,49].

### 3.3 Change analysis

Precipitation and maximum and minimum temperatures were forecast using GCM historical and projected data for four different scenarios. Precipitation changes were expressed as percentages, while Tmax and Tmin were absolute changes. As defined earlier, the temperature and precipitation datasets were examined between the baseline timeframe and three different future periods (near, mid and far).

### 3.4 Trend analysis

The future climatic trends were estimated using the trend-free pre-whitening Mann-Kendall (TFPW-MK) method [13]. The TFPW-MK method is better than the classical Mann-Kendall (MK) test [58,59] and is extensively used by scientists all over the globe to eliminate the effect of serial correlation on trend significance [60,61]. The TFPW-MK can preserve the actual trend in the series [62]. Moreover, Sen's slope (SS) estimator was used to estimate the changing rate [59,63]. Additionally, SS has been routinely used to determine changes in hydro-meteorological data [11,52,64]. Statistical significance was determined using a critical probability value of 0.05 (p < 0.05).

### 3.5 Multi-model ensemble (MME) mean

The multi-model ensemble of 18 GCMs (18-MODEL ENSEMBLE) is calculated by Eq. (5).

(5) MME = 1/n * ∑(i = 1) ^n GCMs_i

where n is the number of GCMs being used in the ensemble, GCMs_i represents the output of the ith GCM, and the symbol ∑(i = 1) ^n denotes the sum of the outputs of all the GCMs being used. The MME mean is the average of the outputs of all the GCMs, and it is used to produce a more reliable estimate of future climate conditions than any single model could provide.

## 4 Results and discussion

### 4.1 Bias-corrected outputs evaluation

**Fig. 2:**
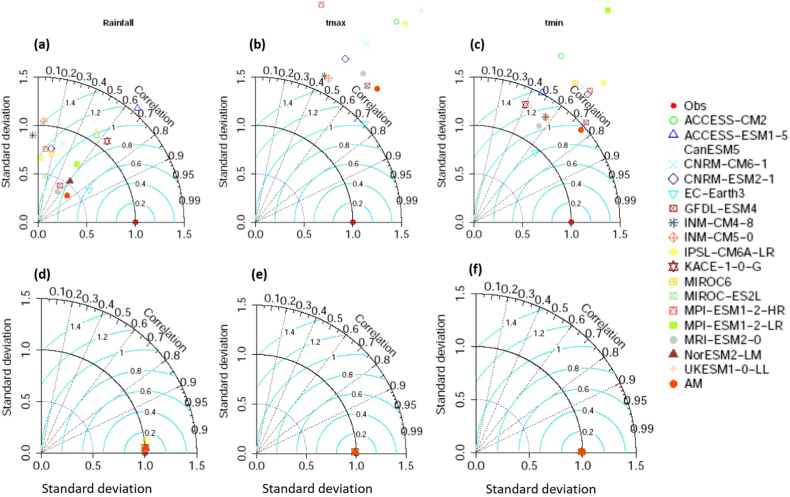
Taylor diagrams for raw (a,b,c) and bias corrected (d,e,f) annual mean precipitation (1st column), Tmax (2nd column), and Tmin (3rd column) of 18 CMIP6 GCMs and their MME mean for the historical period.

**Fig. 3:**
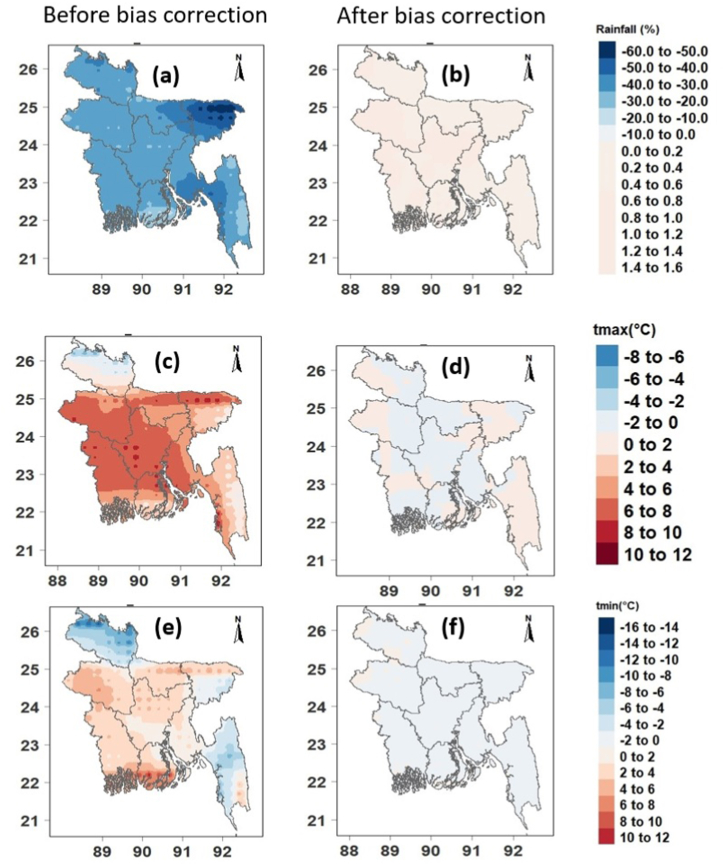
Bias in the MME mean annual precipitation (%) (a) before and (b) after bias correction, Tmax (^◦^C) (c) before and (d) after bias correction, and Tmin (e) before and (f) after bias correction.

Fig. 2 shows the performance of GCM precipitation, Tmax, and Tmin using Taylor diagrams (Fig. 2a–c) and the bias-corrected (Fig. 2d–f) outputs with respect to the observations from 1985 to 2014. For rainfall, all raw model R-values were lower than 0.65 (Fig. 2a). The model's bias-corrected values were higher than 0.99 (Fig. 2d). Before bias-correction, the normalized SDs of rainfall ranged from 0.37 to 1.5. At the same time, it was closer to 1 after bias-correction, suggesting that the rainfall SDs were near to ERA5 SDs. The SQM decreased the centered RMS deviations. Similarly, the normalized SD and R values were all nearer to 1, and the RMS values were low for bias-corrected Tmax and Tmin compared to rows Tmax and Tmin. Overall, all 18 GCMs' performance improved after applying SQM. Fig. 2 also demonstrates that the MME mean of the models was superior to that of individual models in simulating all climate variables when the models were given equal weight in developing the MME. In particular, the MME had higher R-values (>0.99) than individual models for rainfall, Tmax, and Tmin. MME's normalized SDs were closer to the individual models, and MME's centered RMS deviations in the three climatic parameters were lower than the individual models. In general, the MME outperforms individual models. The average MME precipitation, Tmax, and Tmin biases for 1985–2014 prior and post bias correction are shown in Fig. 3. The results suggest that the MME of CMIP6 GCMs had a significant bias before bias correction (Fig. 3a, c, e). For instance, the rainfall over Bangladesh was overestimated but underestimated in the northeastern region (bias >60%), and Tmax and Tmin were underestimated before bias correction (Fig. 3a, c, e). Thus, the SQM technique was employed to adjust the erroneous precipitation estimates, Tmax, and Tmin. The results showed that the biases of all three climate variables were dramatically reduced after corrections using SQM (Fig. 3b, d, and f). The findings indicate that the SQM approach significantly lowered the biases in the CMIP6 GCMs, allowing the findings to be utilized for effective CC impact assessments. Hence, the average MME bias-corrected results were used to investigate predicted future temperature and precipitation changes across Bangladesh from 2015 to 2100.

### 4.2 Air temperature and precipitation projection

#### 4.2.1 Mean monthly projection

**Fig. 4:**
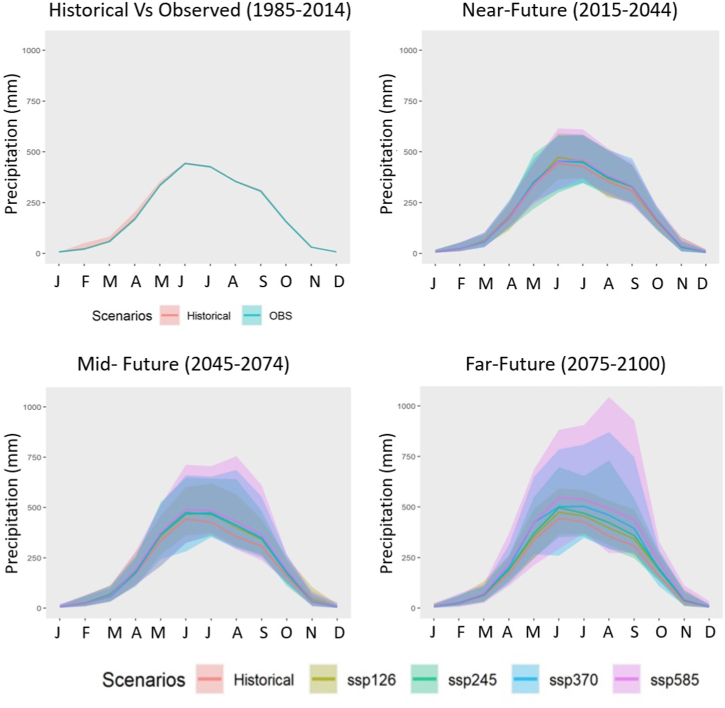
Monthly precipitation (mm) cycle for the historical period and four SSPs in three future periods. The shaded area represents the uncertainty (one inter-model standard deviation) of 18 CMIP6 GCMs.

**Fig. 5:**
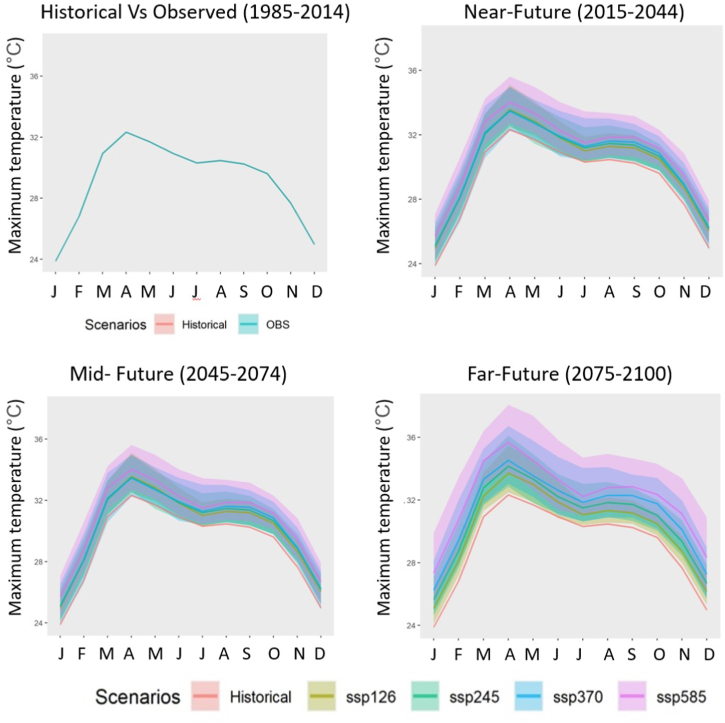
Monthly cycle of Tmax (◦C) for the historical period and four SSPs in three future periods. The shaded area represents the uncertainty (one inter-model standard deviation) of 18 CMIP6 GCMs.

**Fig. 6:**
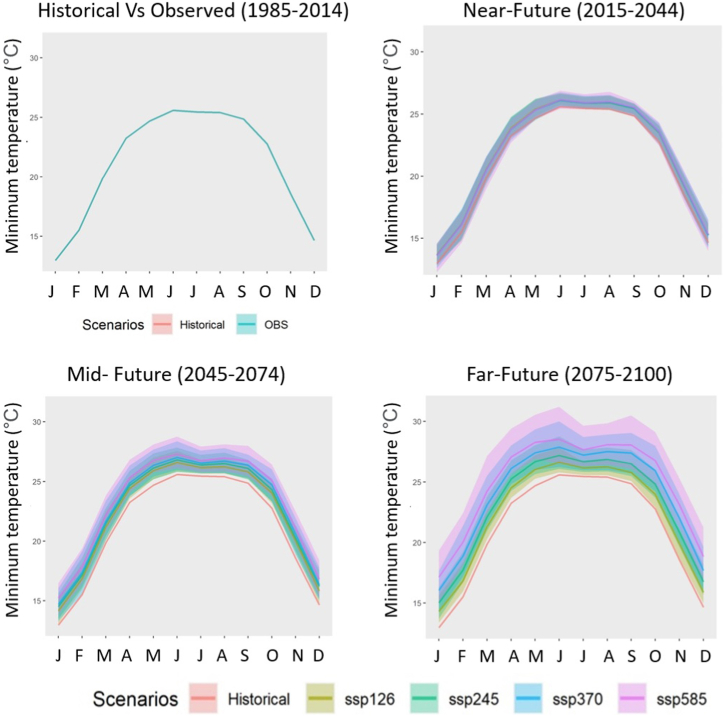
Monthly cycle of Tmin (◦C) for the historical period and four SSPs in three future periods. The shaded area represents the uncertainty (one inter-model standard deviation) of 18 CMIP6 GCMs.

To further understand seasonal variations, the mean changes in precipitation, Tmax, and Tmin for various months were also looked at. All four SSPs were used to examine the expected variations in precipitation, Tmax, and Tmin for various months across three time periods (2015–2044, 2044–2074, and 2075–2100). These changes are presented in Fig. 4, Fig. 5, Fig. 6 for SSP1-2.6, SSP2-4.5, and SSP3-7.0. SSP1-2.6, SSP2-4.5, and SSP3-7.0 all have these alterations seen in Fig. 4, Fig. 5, Fig. 6.

Fig. 4 and Table 1 depicts a rise in precipitation (%) in certain future periods and scenarios in all months other than November through March. For all SSPs, the far future rise in rainfall was predicted to be greater than the increases in the near- and far futures. In January, the precipitation values for SSP2-4.5 and SSP3-7.0 are expected to fall by 6.65 and 13.92%, respectively, in the mid-future. In the near future, the precipitation in February is predicted to fall by 10.08% and 11.33% for SSP1-2.6 and SSP5-8.5, respectively. The highest reduction in precipitation for the month of December was anticipated to be 30.70% in the mid-future, according to SSP3-7.0, followed by 20.16% in the far-future, according to SSP2-4.5. In the far future, SSP5-8.5 will see an increase in precipitation of more than 45% between September and November.

**Table 1:** Changes in monthly precipitation (%) in future periods for four SSPs.

Future period	Scenario	Month											
		Jan	Feb	Mar	Apr	May	Jun	Jul	Aug	Sep	Oct	Nov	Dec
NF	SSP126	2.20	−10.08	5.14	9.82	0.39	7.50	4.34	2.48	3.57	0.55	10.07	−7.33
	SSP245	6.96	3.97	−3.17	4.28	5.32	3.54	3.17	1.88	5.51	1.32	−7.21	−19.77
	SSP370	2.78	−0.25	−4.70	5.84	1.63	2.92	3.54	3.03	5.62	6.99	0.90	−15.45
	SSP585	−0.33	−11.33	2.42	6.10	0.89	1.05	5.03	7.34	5.71	8.00	12.42	2.81
MF	SSP126	−5.29	14.77	15.89	7.21	7.10	1.97	8.11	9.11	10.05	11.96	23.71	−7.75
	SSP245	−6.65	11.29	11.97	5.38	9.48	7.04	4.83	10.46	9.12	9.91	−3.48	−11.82
	SSP370	−13.92	−3.25	−7.54	4.47	7.66	4.89	9.16	8.97	9.13	10.81	4.31	−30.70
	SSP585	−9.18	8.33	4.01	9.14	12.02	10.12	8.00	14.57	11.95	17.06	15.11	−12.24
FF	SSP126	28.04	17.32	12.36	10.54	4.45	6.71	3.78	10.74	7.96	16.65	−0.36	−1.25
	SSP245	−4.81	8.59	7.63	14.63	6.27	10.98	7.37	13.39	13.17	22.12	20.70	−13.09
	SSP370	1.62	2.76	18.05	12.10	23.09	9.37	11.53	17.31	17.68	20.20	15.63	−20.16
	SSP585	8.50	23.08	18.16	26.86	22.60	19.39	16.15	22.89	26.88	45.46	24.36	−11.79

Fig. 5 and Table 2 illustrate the anticipated changes in mean monthly maximum temperature (Tmax) for the near, mid, and far futures under the SSP1-2.6, SSP2-4.5, SSP3-7.0, and SSP5-8.5 scenarios. Tmax was projected to increase in all months for all futures and SSPs. The greatest increase in Tmax would be in February by 0.33–0.64 °C in the near, 1.27–1.97 °C in the mid and 1.31–3.88 °C in the far future for all SSPs, while relatively less increase in the June–August. However, a larger increase in Tmax will be in the far future than in the near and mid future for all SSPs.

**Table 2:** Changes in monthly Tmax (°C) in three future periods for four SSPs.

Future period	Scenario	Month											
		Jan	Feb	Mar	Apr	May	Jun	Jul	Aug	Sep	Oct	Nov	Dec
NF	SSP126	0.52	0.64	0.52	0.53	0.55	0.39	0.37	0.52	0.59	0.52	0.52	0.58
	SSP245	0.34	0.33	0.34	0.21	0.25	0.28	0.35	0.44	0.53	0.42	0.42	0.42
	SSP370	0.36	0.34	0.31	0.15	0.22	0.27	0.34	0.49	0.54	0.43	0.37	0.37
	SSP585	0.33	0.35	0.29	0.20	0.32	0.33	0.34	0.50	0.57	0.46	0.46	0.42
MF	SSP126	1.08	1.27	1.17	1.23	1.15	0.90	0.71	0.81	0.95	0.89	1.00	1.08
	SSP245	1.24	1.33	1.14	1.18	1.05	0.93	0.88	1.00	1.13	1.06	1.22	1.26
	SSP370	1.20	1.29	1.24	1.15	1.00	0.99	0.97	1.15	1.31	1.25	1.24	1.21
	SSP585	1.85	1.97	1.90	1.71	1.62	1.35	1.21	1.39	1.61	1.58	1.83	1.74
FF	SSP126	1.21	1.31	1.33	1.39	1.31	0.94	0.76	0.85	0.94	0.87	1.04	1.19
	SSP245	1.77	1.92	1.86	1.84	1.65	1.28	1.19	1.37	1.49	1.42	1.68	1.71
	SSP370	2.37	2.60	2.40	2.22	1.87	1.70	1.55	1.82	2.05	2.16	2.44	2.31
	SSP585	3.46	3.88	3.59	3.36	2.85	2.38	1.91	2.32	2.62	2.75	3.45	3.38

Fig. 6 and Table 3 illustrate the anticipated change in average monthly Tmin for the near, mid, and far futures for different SSPs. In all months, the estimated monthly Tmin increased for all periods and SSPs. November would expose to the biggest increase in Tmin by 0.73–0.82 °C in the near, 1.45–2.53 °C in the mid, and 1.34–4.57 °C in the far future for all SSPs, whereas June–August would experience a relatively smaller increase. However, like Tmax, Tmin will increase more in the far future than other periods for all SSPs.

**Table 3:** Changes in monthly Tmin (°C) in three future periods for four SSPs.

Future period	Scenario	Month											
		Jan	Feb	Mar	Apr	May	Jun	Jul	Aug	Sep	Oct	Nov	Dec
NF	SSP126	0.70	0.63	0.73	0.64	0.71	0.55	0.41	0.53	0.59	0.76	0.73	0.66
	SSP245	0.66	0.60	0.68	0.54	0.65	0.51	0.41	0.51	0.59	0.74	0.73	0.60
	SSP370	0.64	0.60	0.66	0.54	0.64	0.55	0.43	0.56	0.63	0.83	0.81	0.60
	SSP585	0.63	0.58	0.66	0.55	0.64	0.56	0.44	0.60	0.67	0.82	0.82	0.67
MF	SSP126	1.19	1.35	1.34	1.18	1.22	1.00	0.70	0.84	0.95	1.28	1.45	1.18
	SSP245	1.53	1.65	1.64	1.43	1.45	1.21	0.92	1.10	1.24	1.55	1.68	1.56
	SSP370	1.72	1.85	1.81	1.64	1.66	1.42	1.07	1.32	1.52	1.96	2.01	1.68
	SSP585	2.27	2.33	2.36	2.08	2.07	1.70	1.29	1.55	1.86	2.34	2.53	2.19
FF	SSP126	1.35	1.32	1.46	1.29	1.34	1.04	0.71	0.85	0.94	1.20	1.34	1.24
	SSP245	2.05	2.22	2.28	2.02	1.98	1.59	1.22	1.47	1.65	2.05	2.28	2.11
	SSP370	3.08	3.36	3.25	2.87	2.72	2.29	1.76	2.11	2.53	3.16	3.46	3.05
	SSP585	4.17	4.53	4.40	3.82	3.58	2.94	2.20	2.68	3.19	3.99	4.57	4.20

#### 4.2.2 Annual temporal projected changes

**Fig. 7:**
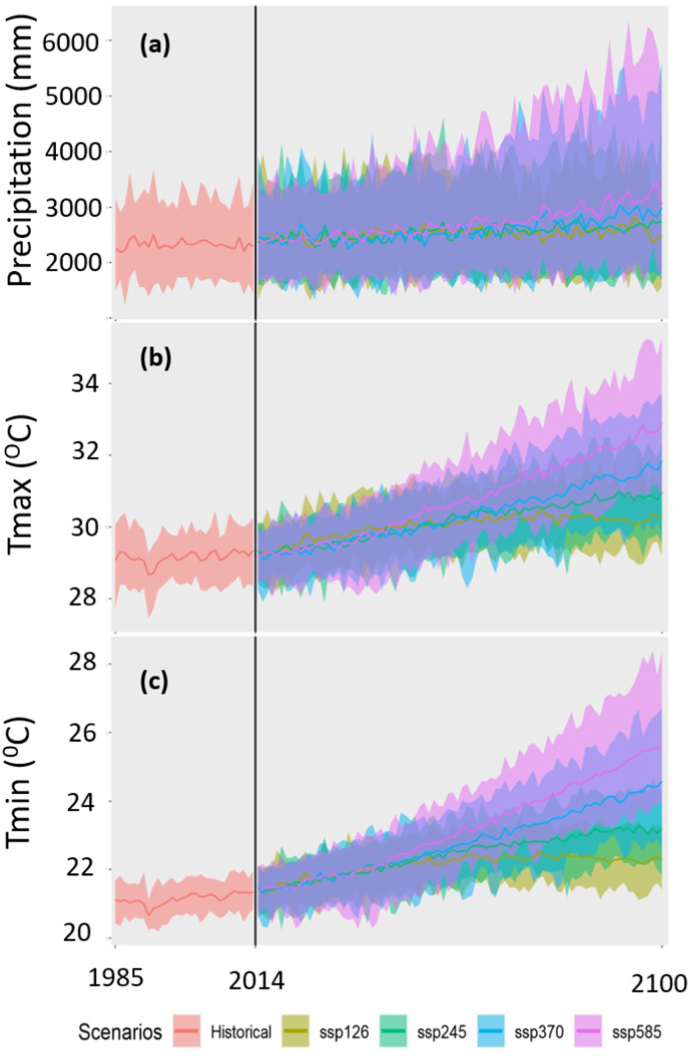
Areal average annual (a) precipitation (mm), (b) Tmax (◦C), and (c) Tmin (◦C) in Bangladesh from 1985 to 2100. Historical simulations (red), SSP12.6 (light green), SSP24.5 (green), SSP370 (blue), and SSP5-8.5 (pink) from the mean (solid line) of the MME, with one inter-model standard deviation (shaded area).

Fig. 7 displays long-term projections for area-averaged precipitation, Tmax, and Tmin across Bangladesh. Precipitation and air temperature exhibited a variable rising trend from 2015 to 2100 for different scenarios. Fig. S1 of the supplementary material shows the change in annual precipitation and air temperature (Tmax and Tmin) compared to 1985–2014.

The annual precipitation changes across Bangladesh from 2015 to 2100 was: 1.75%–21.06% for SSP1-2.6, −2.6%–19.3% for SSP2-4.5, −3.3%–30.3% for SSP3-7.0, and 0.6%–47.9% for SSP5–8.5 (Fig. S1a). In the far future (2075–2100), the mean precipitation across Bangladesh was projected to increase most by 30.9% for SSP5-8.5, followed by 21.1% for SSP2-4.5 and 13.6% for SSP3-7.0, and the least projected precipitation increase was 9.5% for SSP1-2.6.

From 2015 to 2100, the average annual Tmax and Tmin went up almost the same amount in Fig. 7b and c. Both temperatures were projected to rise continuously for SSP3-7.0 and SSP5-8.5. However, the temperatures were predicted to rise slightly just before the mid-century and to fall gently later (around 2075) under SSP1-2.6. (Fig. S1 b, c). SSP1-2.6 was the only scenario where the temperature increases by 2100 were less than 2 °C, as shown in Fig. 5b, c. After 2085, it is expected that the increases in Tmax and Tmin for SSP2-4.5 would still be greater than 2 °C. For SSP5-8.5, the average Tmax (Tmin) was predicted to rise more than 2 °C by around 2070 (2060), suggesting that Tmin will rise faster than Tmax. Furthermore, the variations in projected air temperature increases between the various scenarios were comparatively lower until 2055 for both Tmax and Tmin but increased gradually after that. In the far future, the average annual Tmax (Tmin) was projected to rise by 1.10 (1.17 °C), 1.60 (1.9 °C), 2.12 (2.80 °C), and 2.99 (3.6 °C) from lower to higher SSPs. Fig. 7 suggests a noticeable variability among the 18 models, indicating large uncertainty in the projection of air temperature and precipitation. The shaded regions indicate an increase in projection uncertainty with time.

#### 4.2.3 Spatial variability in projected changes

**Fig. 8:**
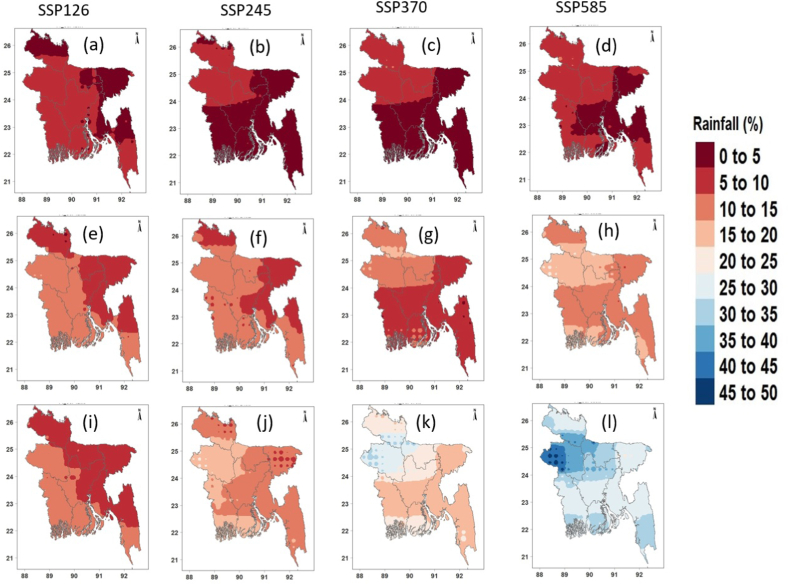
Changes (%) in annual mean precipitation in the (a–c) near, (d–f) mid, and (g–i) far futures for four SPPs.

**Fig. 9:**
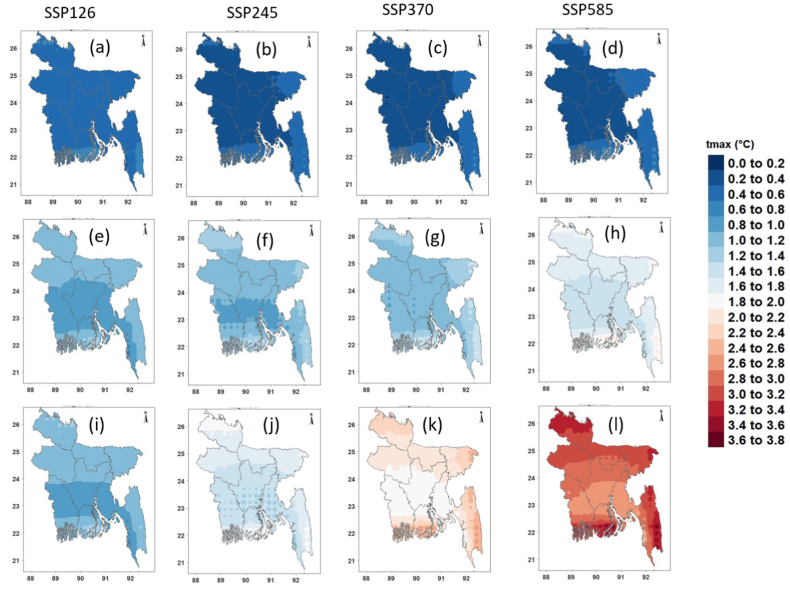
Changes (°C) in annual mean Tmax in the (a–c) near, (d–f) mid, and (g–i) far futures for four SPPs.

**Fig. 10:**
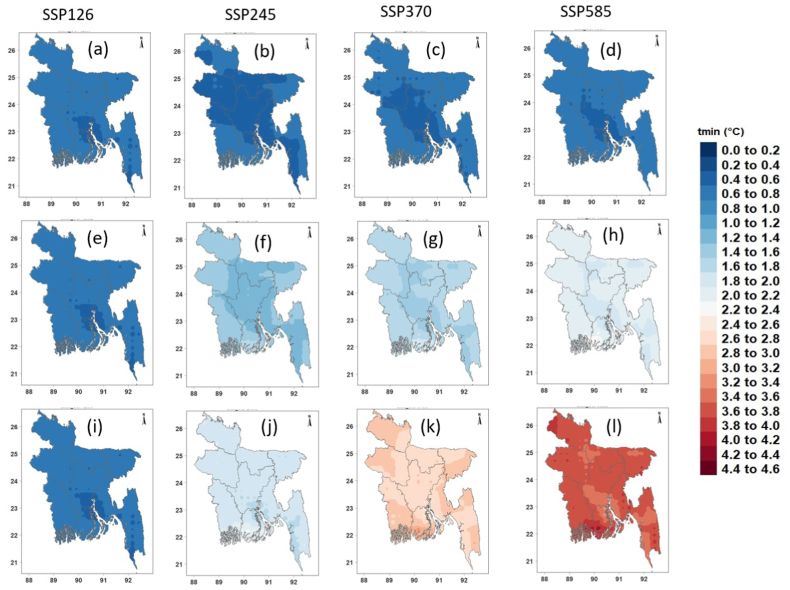
Changes (°C) in annual mean Tmin in the (a–c) near, (d–f) mid, and (g–i) far futures for four SPPs.

Fig. 8, Fig. 9, Fig. 10 show the spatial changes in the projected mean annual precipitation, Tmax and Tmin in the future for four SSPs. The projected annual mean precipitation was predicted to rise over Bangladesh in all periods and scenarios relative to the reference timeframe (Fig. 8a–l). Notably, precipitation was projected to rise by >12% in the near future (Fig. 8a–d) and by 4–23% in the mid-future (Fig. 8e–h) for four SSPs. The most significant rise in precipitation was projected in the far future at 22–50% for SSP5-8.5, followed by 15–36% for SSP3-7.0, 8–21% for SSP2-4.5, and 5–14% for SSP1-2.6 (Fig. 8i–l). The most significant rise in average annual precipitation was anticipated for northwestern Bangladesh.

Fig. 9 shows the geographical variability of anticipated annual average Tmax changes for four distinct SSPs. In every scenario, the northern and coastal regions are anticipated to experience a greater increase in temperature. The annual mean Tmax was expected to rise by 0.86, 0.63, 0.65, and 0.78 °C in the near future (Fig. 9a–d) and by 1.43, 1.53, 1.67, and 2.25 °C in the mid future for lower to higher SSPs, respectively (Fig. 9e–h). In the far future, Tmax is expected to grow by 1.46, 2.03, 2.82, and 3.79 °C for four SSPs, respectively (Fig. 9i-l). In the far future, the southern coastal area was predicted to have the greatest rise in Tmax (3.5 °C).

Similar to Tmax, Tmin showed a sharp rise across Bangladesh for all four scenarios (Fig. 10). The rise in Tmin was predicted to be higher than Tmax and was projected to rise by 0.84, 0.78, 0.80, and 0.82 °C in the near future (Fig. 10a–d), 1.50, 1.82, 2.04, and 2.51 °C in the mid-future (Fig. 10e–h), and 1.56, 2.38, 3.34, and 4.26 °C in the far, and 2.51 °C in the mid-future (Fig. 10e–h), and 1.56, 2.38, 3.34, and 4.26 °C in the far future like Tmax, the greatest rise in average annual Tmin was projected for SSP5-8.5. The largest rise in Tmin (≥4 °C) was projected for the southern coastal region of Bangladesh.

#### 4.2.4 Future trends

MME annual average precipitation, Tmax, and Tmin trends over the period (2015–2100) for various scenarios were investigated using TFPW-MK tests and SS analysis (Table 4). There was a statistically significant rising trend (p< 0.01) in all three variables. The annual rainfall was projected to rise by 16.88, 35.28, 62.45- and 93.59-mm decade^−1^ for SSP1-2.6 to SSP5-8.5, respectively. On the other hand, the mean annual Tmax (Tmin) was projected to rise by 0.10 (0.09), 0.21 (0.22), 0.30 (0.37), and 0.44 (0.53) °C decade^−1^ for SSP1-2.6 to SSP5-8.5, respectively. For each scenario of emissions, the findings show that precipitation and air temperature would rise in Bangladesh.

**Table 4:** Trends in the MME mean annual rainfall, Tmax and Tmin from 2015 to 2100.

Climatic variable	SSP126		SSP245		SSP370		SSP585	
	Z	Slope	Z	Slope	Z	Slope	Z	Slope
Precipitation	3.62	16.88	9.75	35.28	5.61	62.45	7.90	93.59
Tmax	3.32	0.10	6.45	0.21	9.01	0.30	5.37	0.44
Tmin	2.76	0.09	5.78	0.22	5.28	0.37	4.57	0.53

Note: |Z| >2.58 indicates significance at 0.01. Sen's slope estimator uses the following units for precipitation, Tmax, and Tmin: mm decade^−1^, °C decade^−1^, and °C decade^−1^, respectively.

**Fig. 11:**
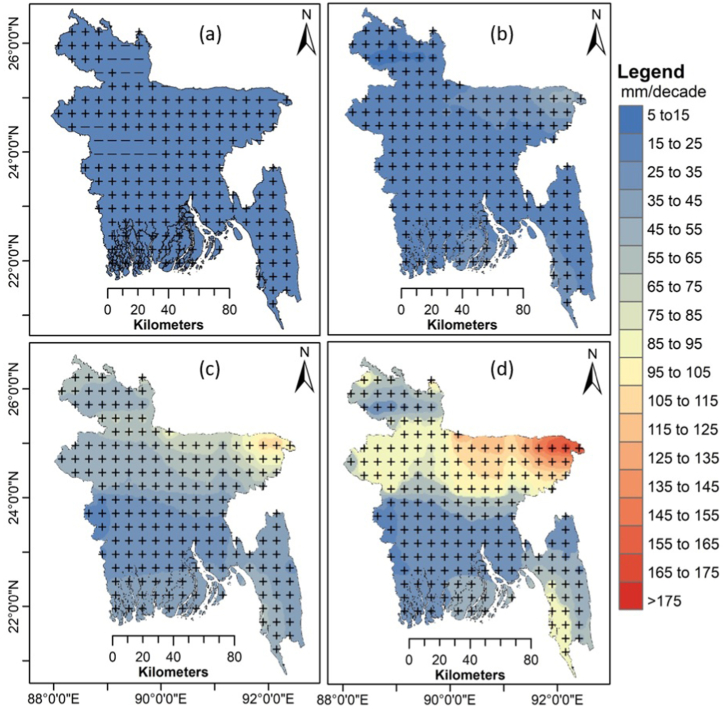
Spatial distribution of the trends in MME mean annual precipitation (in mm decade^−1^) during 2015–2100 for (a) SSP1-2.6, (b) SSP2-4.5, (c) SSP3-7.0 and (d) SSP5-8.5. The "+" indicates a significant trend at p < 0.05.

Fig. 11 shows spatial patterns in MME annual precipitation trends from 2015 to 2100 for the four SSPs. The precipitation was projected to increase significantly for SSP1-2.6 over most of Bangladesh (p < 0.05) (Fig. 11a). With greater emission levels, more regions of Bangladesh will experience a statistically significant upward precipitation trend. A significantly increasing rainfall trend was projected over the whole of Bangladesh for SSP2-4.5 (Fig. 11b), SSP3-7.0 (Fig. 11c), and SSP5- 8.5 (Fig. 11d). The spatial distribution of the changes revealed that the increased annual precipitation would steadily increase from the south to the north of Bangladesh, especially northeastern regions.

**Fig. 12:**
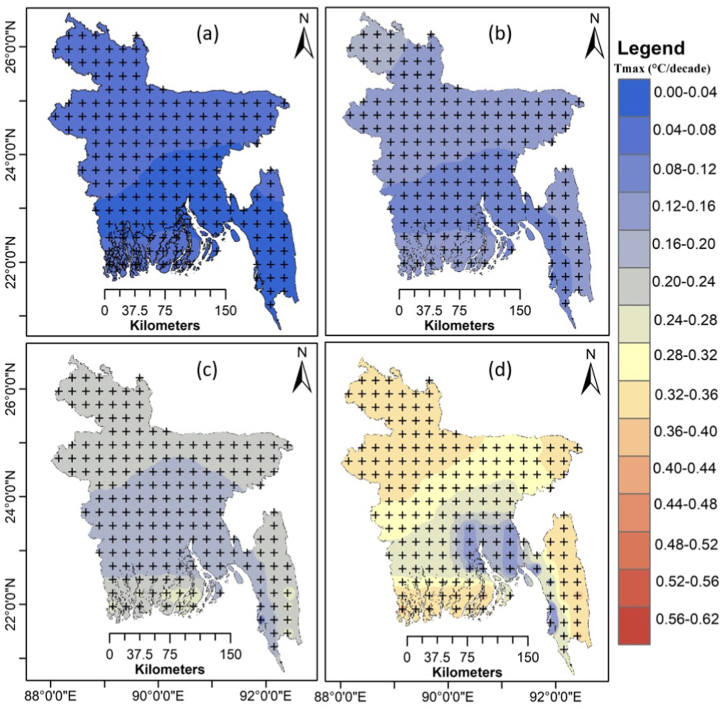
Spatial distribution of the trends in MME mean Tmax (°C decade^−1^) during 2015–2100 for (a) SSP1-2.6, (b) SSP2-4.5, (c) SSP3-7.0 and (d) SSP5-8.5. The "+" indicates a significant trend at p < 0.05.

Fig. 12 shows the variation in average annual Tmax trends around the country for the four SSP scenarios from 2015 to 2100. The projected Tmax displayed a significant increasing trend across all of Bangladesh over the twenty-first century for all SSPs. The Tmax demonstrated a comparatively lower rate of warming (<0.15 °C decade^−1^) across Bangladesh for SSP1-2.6 (Fig. 12a). Still, the warming was more rapid (0.13–0.26 °C decade^−1^) for SSP2-4.5 (Fig. 12b) and even more rapid (0.22–0.38 °C decade^−1^) for SSP3-7.0 (Fig. 12c). The highest rising rate was predicted for SSP5-8.5 by 0.32–0.52 °C decade^−1^ over most of Bangladesh (Fig. 12d).

**Fig. 13:**
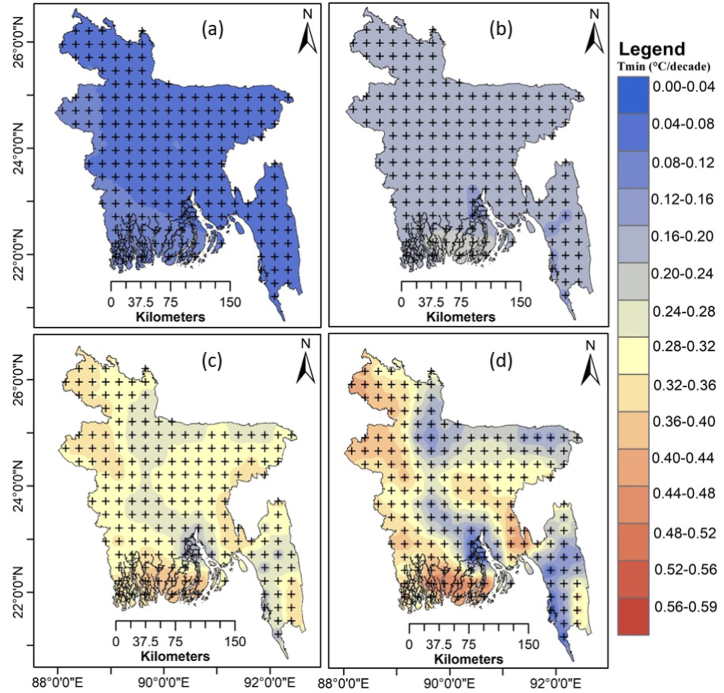
Spatial distribution of the trends in MME mean Tmin (°C decade^−1^) during 2015–2100 for (a) SSP1-2.6, (b) SSP2-4.5, (c) SSP3-7.0 and (d) SSP5-8.5. The "+" indicates a significant trend at p < 0.05.

The spatial distribution of the annual average Tmin trends for 2015–2100 for four SSPs is shown in Fig. 13. The projected Tmin exhibited a significant increasing trend (p < 0.05) over Bangladesh during 2015–2100. The warming rates of the mean annual Tmin were also greater for greater greenhouse gas emission levels. SSP1-2.6 provided the least rate of increase for Tmin (°C decade^−1^) across the country (Fig. 13a). A more rapid warming of 0.19–0.28 °C decade^−1^ was projected for SSP2-4.5 (Figs. 13b) and 0.33–0.44 °C decade^−1^ for SSP3-7.0 (Fig. 13c). The largest rate of increase for Tmin was 0.47–0.6 °C decade^−1^ for SSP5-8.5 (Fig. 13d). The highest increased was demonstrated in the western part and southern coastal region of Bangladesh.

#### 4.2.5 Seasonal analysis

Table 5 details the projected changes in seasonal average precipitation, Tmax, and Tmin from the historical epoch over three different durations.

**Table 5:** Changes in seasonal precipitation (%), Tmax (°C) and Tmin (°C) for four SSPs in three future periods.

Variable	Scenario	Pre-monsoon			Monsoon			Post-monsoon			Winter		
		Near	Mid	Far	Near	Mid	Far	Near	Mid	Far	Near	Mid	Far
Precipitation	SSP1-2.6	3.71	8.05	7.10	4.67	6.96	7.08	2.12	13.90	13.84	−7.02	6.04	15.62
	SSP2-4.5	4.12	8.51	8.92	3.45	7.63	10.97	−0.09	7.70	21.89	−0.36	2.86	1.37
	SSP3-7.0	2.23	5.11	19.27	3.66	7.87	13.48	5.98	9.74	19.45	−2.80	−11.12	−2.24
	SSP5.-8.5	2.61	10.32	23.41	4.55	10.93	20.80	8.73	16.74	41.98	−6.17	0.52	12.88
Tmax	SSP1-2.6	0.50	1.10	1.29	0.46	0.82	0.84	0.54	0.94	0.93	0.58	1.07	1.16
	SSP2-4.5	0.29	1.07	1.70	0.37	0.95	1.26	0.47	1.11	1.47	0.41	1.26	1.71
	SSP3-7.0	0.26	1.09	2.00	0.39	1.04	1.71	0.41	1.27	2.25	0.35	1.24	2.32
	SSP5.-8.5	0.33	1.76	3.22	0.40	1.33	2.18	0.49	1.65	2.98	0.48	1.86	3.44
Tmin	SSP1-2.6	0.68	1.19	1.27	0.52	0.84	0.88	0.77	1.33	1.28	0.66	1.21	1.24
	SSP2-4.5	0.64	1.44	1.97	0.52	0.84	0.88	0.76	1.59	2.12	0.61	1.58	2.10
	SSP1-2.6	0.63	1.60	2.74	0.51	1.10	1.45	0.79	1.97	3.27	0.61	1.74	3.12
	SSP2-4.5	0.68	2.15	3.71	0.52	1.28	2.14	0.88	2.39	4.20	0.70	2.28	4.26

##### 4.2.5.1 Precipitation

Monsoon, pre-monsoon, and post-monsoon seasons observed a rise in precipitation, whereas winter seasons experienced a drop across all scenarios and time periods (Table 6). The post-monsoon was predicted to have the greatest precipitation increase. For the SSP5-8.5 scenario, an increase of 16.74% was predicted in the near future, while a significantly higher increase of 41.98% was projected for the far future. The monsoon and post-monsoon precipitations were projected to increase by 20.80% and 23.41% in the far future for SSP5-8.5, respectively. SSP2-4.5 was anticipated to have the smallest increase in monsoon precipitation (3.45%). Precipitation levels after the monsoon (post-monsoon) were predicted to drop by 0.09% for SSP2-4.5 in the near future. The predicted change in winter precipitation was between −7.02% and 15.62% for SSP1-2.6, −0.36% and 2.86% for SSP2-4.5, −2.24% and −11.12% for SSP3-7.0, and between −6.17% and 12.88% for SSP5-8.5. SSP3-7.0 projected the most significant precipitation decrease (11.12%) in the mid-future.

**Table 6:** Raw and bias-corrected GCM projections of precipitation and air temperature across Bangladesh.

Variables	Scenarios	Projection based on raw data			Projection based on bias corrected data		
		Near	Mid	Far	Near	Mid	Far
Rainfall (mm)	SSP126	1699.09	1764.15	1987.46	2455.66	2555.73	2549.82
	SSP245	1685.33	1756.72	1798.71	2431.72	2566.68	2646.37
	SSP370	1692.29	1751.07	1913.36	2439.88	2551.52	2819.63
	SSP585	1697.80	1822.75	2048.15	2462.79	2664.80	3048.79
Tmax (°C)	SSP126	31.14	31.75	30.41	29.67	30.17	30.24
	SSP245	30.94	31.89	32.50	29.51	30.27	30.75
	SSP370	30.91	31.93	33.18	29.50	30.32	31.27
	SSP585	30.95	32.56	34.30	29.53	30.80	32.14
Tmin (°C)	SSP126	22.09	22.66	22.84	21.769	22.27	22.30
	SSP245	22.03	22.98	23.57	21.73	22.54	23.04
	SSP370	22.05	23.25	24.63	21.75	22.76	23.93
	SSP585	22.07	23.742	25.68	21.77	23.17	24.82

##### 4.2.5.2 Temperature

Winter showed the most substantial rise in Tmax and Tmin, whereas the monsoon season showed the least for all future periods and scenarios. The winter Tmax (Tmin) was projected to rise by 1.16 (1.24), 1.71 (2.10), 2.32 (3.12), 3.44 **°**C (4.26 **°**C) for SSP1-2.6 to SSP5-8.5, respectively, in the far future. In contrast, monsoon Tmax (Tmin) was predicted to increase by 0.84 (0.88), 1.26 (1.45), 1.71 (2.14), 2.18 **°**C (2.70 **°**C) for SSP1-2.6 to SSP5-8.5, respectively, in far-future. The pre-and post-monsoon Tmax (Tmin) were projected to rise by 3.22 (3.71) and 2.98 **°**C (4.20 **°**C), respectively, for SSP5-8.5.

#### 4.2.6 The uncertainties of the projected changes

**Fig. 14:**
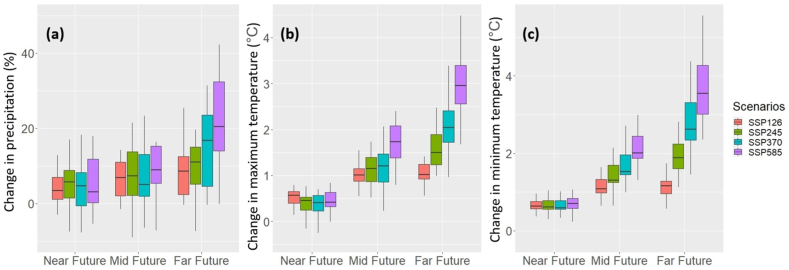
Box-and-whisker graphs illustrating the projected changes in (a) precipitation (%), (b) Tmax (◦C), and (c) Tmin (◦C) throughout Bangladesh in three future periods for SSPs. The box's band represents MME mean. The bottom (top) of the box reflects the MME mean minus (plus) one standard deviation, while the whiskers show the minimum and maximum simulated changes, respectively.

Uncertainties in future precipitation and temperature downscaling or projections are unavoidable [33]. They are the major controlling factor in impact assessment, especially on the local or regional scale [65]. The widely used MME approach to minimize the uncertainties was used herein [66,67]. Box and whisker diagrams were employed to exhibit the ±1 inter-model SD (boxes) and inter-model range (whiskers) for analyzing the uncertainty in the projected air temperature and precipitation (Fig. 14). The findings illustrated that the uncertainty in projected precipitation, Tmax, Tmin, increased over time for all scenarios. This is consistent with the results presented in Fig. 7. For the near and mid futures, the uncertainties in precipitation, Tmax, and Tmin were identical for all scenarios. However, the uncertainties for the SSP5-8.5 were greater than the others in the far future. Moreover, Fig. 14 indicates the MME mean projection has lower uncertainty ranges than each of the 18 bias-corrected models. Despite the advancements, uncertainties remain in the projected air temperature and precipitation. More research is needed to reduce these uncertainties. In reality, a small MME can be chosen by removing those that are deemed unrealistic to reduce the GCM-related uncertainty [68].

#### 4.2.7 Evaluation of before and after bias-corrected data

Table 6 shows the evaluation of the MME mean precipitation, Tmax, and Tmin of the original and bias-corrected projections. The findings suggest that raw GCM rainfall projections are lower than the bias-corrected precipitation projections for the identical period and scenario, whereas Tmax and Tmin projections of raw GCMs are greater than the bias-corrected data. The findings confirm that the CMIP6 GCMs underestimated the precipitation and overestimated Tmax and Tmin for Bangladesh (Section 4.1). This suggests the need for bias correction of climate data for Bangladesh before the change and trend analysis.

The bias-corrected average MME showed a considerable rise in precipitation across Bangladesh for the period 2015–2100. The lowest and greatest increases were projected for SSP1-2.6 and SSP5-8.5, respectively. The Tmax and Tmin were also projected to rise across Bangladesh for all four scenarios. However, the rate of rise was nearly identical for the various scenarios. The air temperature was projected to rise continuously over time for SSP3-7.0 and SSP5-8.5, but it is likely to rise in the near and mid futures and then be steady or slightly drop in the far future for SSP1-2.6. Tmin was projected to rise faster than Tmax for all periods and scenarios, which coincided with the findings of past investigations across Bangladesh using CMIP5 GCMs [69].

## 5 Discussion

### 5.1 Bias-correction and uncertainties

This study presents a comprehensive analysis of temperature and precipitation projections for Bangladesh using CMIP6 GCMs. It provides valuable insights into the expected changes in precipitation, Tmax, and Tmin for Bangladesh, which is crucial for climate impact assessment and long-term adaptation planning. The SQM approach effectively reduced the biases in CMIP6 GCMs, and the bias-corrected output demonstrated a higher agreement with the historical observations. In addition, the MME of the GCMs outperformed the individual models in simulating all climate variables. The consistency of these findings across multiple studies highlights the importance of the SQM technique in climate modeling [21,48,49,70]. Many studies have shown that the MME forecast performance is superior to the forecast of an individual (one-model-based) ensemble prediction systems [[71], [72], [73], [74], [75]]. The findings suggest that MME approach can provide more accurate and robust climate projections, which can inform long-term adaptation planning and decision-making. As CC continues to pose significant challenges to vulnerable regions such as Bangladesh, the use of advanced modeling techniques like SQM is crucial for developing effective adaptation strategies and building climate resilience.

### 5.2 Mean monthly projection

The precipitation is expected to increase in future periods and scenarios considered in all months except for few scenarios in November–March. The rise in rainfall for far future is predicted to be greater than in the near- and mid-futures, which agrees with previous studies where increased rainfall during May–October for RCP4.5 and all three future periods for RCP8.5 were reported [11]. Such increase in rainfall would be beneficial for irrigated crops but could be harmful for excess soil water sensitive crops. Depending on the scenarios and time period during December–January, a decline in rainfall may lead to a rise in irrigation costs in the future. Conversely, the projections for the SSP5-8.5 scenario in the far future indicate that the region could encounter a precipitation surge of more than 45% from September to November. This suggests that the effect of climate change on precipitation could intensify with time, potentially affecting water availability, agricultural productivity, and flood risk in the area.

Although Tmax and Tmin would be increased in all months for all futures and SSPs, the warming is expected to be more pronounced in winter months (December–February). However, Tmin is projected to increase more than Tmax for all periods and scenarios. which is coincided with the findings of past investigations for the MME of CMIP5 GCMs over Bangladesh [18,19,76]. Moreover, both Tmax and Tmin are predicted to rise sharply across Bangladesh, with the coastal areas and northern region expected to experience the greatest increase which is partially coincided with the previous study of [19] using CMIP5 GCMs. As a result, diurnal temperature range (DTR) would decrease in future, that has potential negative consequences in public health and agricultural production [19,77]. Increase in night temperature will augment higher respiratory losses in crops and thus reduced grain yield. Moreover, comfortable temperature zone for growth and development for livestock and fishes would be hampered greatly.

### 5.3 Annual projection

The study showed anticipated increase in precipitation by 9.48%, 13.63%, 21.07%, and 30.90% for SSP1-2.6, SSP2-4.5, SSP3-7.0, and SSP5-8.5, respectively. This result is coincided with the findings of [11]. Using a Multi-Model Ensemble (MME) of 40 CMIP5 Global Climate Models (GCMs), it was projected that Bangladesh may experience an increase in annual rainfall of 2.76–5.98% and 6.98–26.44% under RCP4.5 and RCP8.5 scenarios, respectively.

In addition, the average Tmax (Tmin) is projected to rise by 1.09 (1.17), 1.60 (1.91), 2.12 (2.80), and 2.99 (3.69) °C for the same scenarios in the far future. The rise in both Tmax and Tmin is also projected to be higher in the far future, in line with previous studies. Caesar et al. [17] utilized a 17-member perturbed physics ensemble of projections from a GCM to drive their RCM over South Asia from 1971 to 2099 and reported a projected increase in annual mean temperature by 2.6–4.8 °C by the year 2100, relative to the reference period. Similarly, Alamgir et al. [76] used eight CMIP5 GCMs to perform statistical downscaling over Bangladesh and predicted an increase in temperature by 2.7–4.7 °C under RCP 8.5 by the end of the century. Recently, Islam et al. [19] found that the mean annual maximum temperature over Bangladesh is expected to rise by 0.61 °C and 1.75 °C in the near future and by 0.91 °C and 3.85 °C in the far future for RCP4.5 and RCP8.5, respectively. Also, they predicted that the mean annual minimum temperature would rise by 0.65 °C and 1.85 °C in the near future and by 0.96 °C and 4.07 °C in the far future for RCP4.5 and RCP8.5, respectively, which is in line with what our study found.

The previous study [19] projected an average temperature rise of 3.24–5.77 °C in Bangladesh by the end of the century and suggested that the southwest and south-central regions would experience a greater temperature increase. The current study shows that in all scenarios, Tmax and Tmin are expected to rise across Bangladesh, with the southern coastal region projected to experience the greatest increase in temperature. As southern coastal belt is already vulnerable for agriculture because of water salinity and temperature, further temperature rise will be a great concern for agricultural sustainability in future. The northwestern region, the drier part of Bangladesh, is projected to experience the highest increase in precipitation and thus it might be beneficial for that region in terms of agricultural productivity. It should be noted that the yearly mean precipitation over the northwestern part of Bangladesh is about 1400–1550 mm [78], which is lower across the country. The current study provides more detailed spatial projections of precipitation and temperature changes under different SSPs, which can help in developing adaptation strategies for Bangladesh.

### 5.4 Seasonal projection

The study found that precipitation in Bangladesh is expected to increase in the monsoon, pre-monsoon, and post-monsoon seasons, with the post-monsoon season projected to experience the greatest increase. Winter seasons, on the other hand, are expected to experience a decrease in precipitation across most of the scenarios and time periods. The smallest increase in monsoon precipitation was anticipated for SSP2-4.5, while the most significant decrease in precipitation was projected for SSP3-7.0 in the mid-future. These findings are consistent with previous studies that have also projected an increase in precipitation during monsoon seasons and a decrease in winter precipitation in the Bangladesh due to climate change [11,79]. Increase in precipitation during monsoon might aggravate flash flood situations in many low-lying areas of the country and thus transplanted aman rice, a dominant rainfed rice crop, productivity can be reduced. The greatest increase in temperature was projected for the winter season, while the smallest increase was predicted for the monsoon season. The pre- and post-monsoon seasons were also projected to experience a rise in temperature, with the greatest increase predicted for SSP5-8.5. However, this study provides more detailed and updated projections using a state-of-the-art climate model and a range of different scenarios, allowing for a more comprehensive understanding of the potential impacts of CC on the region.

The results of the study suggest that Bangladesh will experience significant changes in precipitation and temperature patterns in the future. The projected increase in precipitation during the monsoon and post-monsoon seasons may lead to an increased risk of flooding and other related natural disasters [4,19]. These changes in precipitation patterns may have significant implications for agriculture, food security, and water resource management in Bangladesh [21]. On the other hand, the decrease in precipitation during the winter season may lead to water scarcity and other associated problems. Any decrease in surface water will increase dependency on sub-surface water for irrigation and domestic uses. So, preservation of surface water will be one of the adaptation policies for Bangladesh in future. The study's findings also suggest that there will be a significant increase in temperature across all of Bangladesh, with the highest increase predicted for the winter season. This rise in temperature may have adverse effects on public health, particularly for vulnerable populations, such as children and the elderly [2,80]. The projected increase in temperature may also lead to changes in ecosystems, with potential impacts on biodiversity, ecosystem services, and other ecological processes [81].

### 5.5 Implications and limitations of the study

The findings of the research emphasize the pressing requirement for Bangladesh to formulate adaptation strategies to deal with the anticipated alterations in precipitation and temperature patterns. These adaptation strategies should focus on improving water resource management, promoting climate-resilient agricultural practices, enhancing public health infrastructure, and developing climate-resilient infrastructure. The study's findings also emphasize the importance of global efforts to mitigate greenhouse gas emissions and address CC to minimize the adverse impacts on vulnerable countries such as Bangladesh.

This research also highlights the uncertainties associated with precipitation and air temperature projections in Bangladesh. Although the MME method reduced the uncertainties, further efforts are needed to reduce them further. Future studies must focus on improving the accuracy of climate models by incorporating more detailed physical processes, expanding the observational network, and including regional feedback mechanisms. In addition, the study recommends selecting the best models from the CMIP6 for the regional climate across Bangladesh and applying MME based on GCM's performance-based weight for more accurate climate projections.

The study provides crucial information for policymakers, researchers, and practitioners to plan and implement adaptation measures to minimize the negative impacts and harness the positive effects of CC in Bangladesh. The findings underscore the need for continued efforts to improve the accuracy of climate models and reduce uncertainties to make informed decisions for CC impact assessment and long-term adaptation planning.

## 6 Conclusions

This study employed the SQM technique to remove biases from CMIP6 GCMs and generated bias-corrected projections of precipitation, Tmax, and Tmin for Bangladesh. The results showed that the MME of the 18 GCMs outperformed individual GCMs and produced significantly improved bias-corrected projections for all three climate variables. The findings suggest that precipitation is expected to increase in certain future periods and scenarios in all months except November through March. However, the rise in rainfall for the far future is predicted to be greater than the increases in the near- and mid-futures. The study also revealed that Tmax and Tmin are expected to rise across Bangladesh, with the southern coastal region projected to experience the greatest increase in temperature. The projected rise in precipitation is anticipated to be significant in all scenarios, with the most significant increase projected in the far future for SSP5-8.5. Furthermore, the study found that precipitation and air temperature projections in Bangladesh are prone to large uncertainties, highlighting the need for further studies to minimize uncertainties in projections for CC impact assessment.

Overall, the results of this study have significant implications for climate impact assessment and long-term adaptation planning in Bangladesh. The findings can be used to inform policy decisions and guide strategies to mitigate and adapt to the effects of CC in the country. However, more research is required to reduce the uncertainties in climate projections further and enhance our understanding of the complex interactions between different climatic variables in the region.

## Funding statement

This research did not receive any specific grant from funding agencies in public, commercial, or not-for-profit sectors.

## Data availability

Data will be made available on request.

## Authors contributions

Mohammad Kamruzzaman: Conceived and designed the experiments; Performed the experiments; Analyzed and interpreted the data; Contributed reagents, materials, analysis tools or data; Wrote the paper.

Shahriar Wahid: Conceived and designed the experiments; Analyzed and interpreted the data; Contributed reagents, materials, analysis tools or data; Wrote the paper.

Shamsuddin Shahid and Jatish Chandra Biswas: Analyzed and interpreted the data.

Edris Alam, Mohammed Mainuddin, H. M. Touhidul Islam, Jeapil Cho, Md. Mizanur Rahman, and Kelly R. Thorp: Analyzed and interpreted the data; Contributed reagents, materials, analysis tools or data.

## Declaration of competing interest

The authors declare that they have no known competing financial interests or personal relationships that could have appeared to influence the work reported in this paper.

## Figures and Tables

**figs1:**