# Supplementary material for: Predicted changes in future precipitation and air temperature across Bangladesh using CMIP6 GCMs
Source: Heliyon. 2023 May 13;9(5):e16274. doi: 10.1016/j.heliyon.2023.e16274 (PMC10205770; doi:10.1016/j.heliyon.2023.e16274)
Supplement: Table S1 [file mmc1.docx]

**Table 1. Employed CMIP6 models, their developing centers, and spatial resolution (km)**

| **Sl**  **No.** | **Models** | **Modeling Center** | **Spatial**  **Resolution** |
| --- | --- | --- | --- |
| 1 | ACCESS-CM2 | Australian Community Climate and Earth-System Simulator | 192 × 144 |
| 2 | ACCESS-ESM1-5 | Australian Community Climate and Earth-System Simulator | 192 × 144 |
| 3 | CanESM5 | Canadian Earth System Model | 128 × 64 |
| 4 | CNRM-CM6-1 | National Centre for Meteorological Research, France | 256 × 128 |
| 5 | CNRM-ESM2-1 | National Centre for Meteorological Research, France | 256 × 128 |
| 6 | EC-Earth3 | EC-Earth Consortium | 512 × 256 |
| 7 | GFDL-ESM4 | NOAA/ Geophysical Fluid Dynamics Laboratory, USA | 288 × 180 |
| 8 | INM-CM4-8 | Institute for Numerical Mathematics, Russia | 180 × 120 |
| 9 | INM-CM5-0 | Institute for Numerical Mathematics, Russia | 180 × 120 |
| 10 | IPSL-CM6A-LR | Institut Pierre Simon Laplace, France | 144 × 143 |
| 11 | KACE-1-0-G | NIMS-KMA/ South Korea | 192 × 144 |
| 12 | MIROC6 | Atmosphere and Ocean Research Institute (The University of Tokyo), National Institute for Environmental Studies, and Japan Agency for Marine-Earth Science and Technology, Japan | 256 × 128 |
| 13 | MIROC-ES2L | AMSTEC (Japan Agency for Marine-Earth Science and Technology), AORI (Atmosphere and Ocean Research Institute, The University of Tokyo), NIES (National Institute for Environmental Studies), and R-CCS (RIKEN Center for Computational Science), Japan | 128 × 64 |
| 14 | MPI-ESM1-2-LR | Max Planck Institute for Meteorology, Germany | 192 × 96 |
| 15 | MPI-ESM1-2-HR | Max Planck Institute for Meteorology, Germany | 384 × 192 |
| 16 | MRI-ESM2-0 | Meteorological Research Institute, Japan | 320 × 160 |
| 17 | NorESM2-LM | Norwegian Climate Center, Norway | 144 × 96 |
| 18 | UKESM1-0-LL | UK Met Office Hadley Office, UK | 192 × 144 |
